# The Relative Abundance of Benthic Bacterial Phyla Along a Water-Depth Gradient in a Plateau Lake: Physical, Chemical, and Biotic Drivers

**DOI:** 10.3389/fmicb.2019.01521

**Published:** 2019-07-10

**Authors:** Kaiyuan Wu, Wenqian Zhao, Qian Wang, Xiangdong Yang, Lifeng Zhu, Ji Shen, Xiaoying Cheng, Jianjun Wang

**Affiliations:** ^1^School of Environment and Civil Engineering, Jiangnan University, Wuxi, China; ^2^State Key Laboratory of Lake Science and Environment, Nanjing Institute of Geography and Limnology, Chinese Academy of Sciences, Nanjing, China; ^3^School of Biological Sciences, Nanjing Normal University, Nanjing, China; ^4^University of Chinese Academy of Sciences, Beijing, China

**Keywords:** bacteria, phyla, relative abundance, water depth, biotic effects, abiotic effects

## Abstract

Water-depth biodiversity gradient, one of the typical biogeographical patterns on Earth, is understudied for bacteria in freshwater ecosystems, and thus left the underlying mechanisms poorly understood especially for benthic bacteria. Here, we investigated the water-depth distribution of surface sediment bacterial phyla and their driving factors in Lake Lugu, a plateau lake in Southwest China. Our results revealed that the relative abundance of 11 dominant bacterial phyla showed various water-depth patterns, such as increasing, decreasing, hump-shaped, and U-shaped patterns. These patterns across phyla were consistent with their different niche positions of water depth, while the occupancy-abundance relationships were not dependent on phylum attributes. Consistently, phylum abundance was best explained by water depth; other physical and chemical factors, such as metal ion concentrations, SiO_2_, and pH, can also explain the variations in some bacterial phyla. Chemical variables were the main drivers of the dominant bacterial phyla. However, biotic variables also showed substantial importance for some phyla, such as Planctomycetes, Actinobacteria, and WS3. This work could provide new insights into the general water-depth patterns and underlying mechanisms of the relative abundance of bacterial phyla in freshwater ecosystems.

## Introduction

Biogeographical patterns of biodiversity along gradients, such as those of latitude, elevation, and water depth, are among the most widely studied topics in ecology (Zintzen et al., 2017), and researchers have described the latitudinal and elevational patterns in biodiversity in a variety of ecosystems across the globe (Fuhrman et al., 2008; Wang et al., 2011; Shi et al., 2014). Similar to the more commonly studied latitudinal and elevational gradients, water-depth gradient show complex changes in various environmental attributes and thus impose strong environmental filtering on the aquatic microbial community (Bryant et al., 2012). The phylogenetic and physiological diversity of microbial phyla, especially those of bacteria, is considerably greater than that of animal and plant phyla (Prosser et al., 2007; Yeh et al., 2019). Furthermore, benthic bacteria play an important role in organic matter transformation and in the biogeochemical cycling of major elements such as nitrogen, phosphorus, sulfur, and iron (Martins et al., 2011; Cheng et al., 2014). Knowledge of benthic bacterial phyla distribution in aquatic ecosystems is of great significance and can provide novel perspectives regarding water-depth biodiversity patterns among microorganisms and their potential drivers and further contribute to the sustainable management of water resources. Nonetheless, as most studies focus on marine waters (Agogue et al., 2011; Bryant et al., 2012; Walsh et al., 2016), the general patterns and driving mechanisms of bacterial communities along water-depth gradients have been less studied for freshwater ecosystems.

In recent years, the niche position and niche breadth hypotheses have been used to investigate how niche characteristics account for species distribution and abundance (Brandle and Brandl, 2001). Among these two referenced niche parameters, niche position indicates the availability of habitat and resources (Teittinen et al., 2018). Some species have marginal niche positions at the very edges of the environmental ranges, while other species have nonmarginal niche positions that exist under average environmental conditions (Vilmi et al., 2019). Niche position, rather than niche breadth, is the chief predictive factor of species distributions, such as those of stream insects (Heino and de Mendoza, 2016). In addition, previous research has provided strong evidence that the occupancy and abundance relationships of species tend to be positively and often strongly linked with each other in a variety of ecosystems (Gaston et al., 2000; Foggo et al., 2007; Zuckerberg et al., 2009). Niche position could be a strong predictor of species variation in terms of occupancy and abundance. For instance, niche position is negatively associated with mean abundance in diatom and macroinvertebrate species, and the occupancy-abundance relationship clearly differs across taxonomic groups (Vilmi et al., 2019). At the community level, niche position and occupancy are related to the abundance distribution, but whether such a relationship exists at phylum level remains unexplored.

Traditional niche-based theory asserts that the relative abundances of species are determined by abiotic and biotic factors, such as environmental conditions, habitat heterogeneity, and species interactions (Dumbrell et al., 2010; Liao et al., 2016). Most studies have shown that local environmental conditions, such as physical and chemical attributes, can affect microbial community composition and species diversity in lake ecosystems and the biogeochemical processes they mediate (Staley et al., 2015; Qin et al., 2016). The bacterial community can be shaped by a variety of environmental factors, such as water depth, nitrogen, phosphorus, pH, and pollution (Brown et al., 2009; Haller et al., 2011; Song et al., 2012; Liu et al., 2015b; Zhang et al., 2019). In addition, biotic interactions could also affect microbial community structure. However, rarely considered are the important ecological processes associated with biotic variables, such as the community composition and diversity of other organisms that feed on bacteria (Langenheder et al., 2017). Ecosystems are constructed around interaction webs, such as predation, mutualism, competition, and host-parasite interactions, that connect every species to many others at an array of spatial scales (Estes et al., 2011; Amin et al., 2012). For example, viral abundance is typically strongly correlated with bacterial abundance in marine systems (Clasen et al., 2008; Hanson et al., 2017). In the soil, fungi and bacteria have an effect on the formation of each otherʼs community structure (Singh et al., 2009). Moreover, biotic attributes such as the composition of other taxonomic groups are generally better predictors of species diversity in streams than other environmental variables (Johnson and Hering, 2010).

Here, we explored the water-depth patterns and underlying drivers of the relative abundance of bacterial phyla in the surface sediments of Lake Lugu, a typical deep plateau lake system located in Southwest China. This lake has a water depth of 93.5 m and an area of 50.5 km^2^. We had the four following objectives. First, we examined the water-depth patterns in terms of the relative abundance of benthic bacterial phyla. Second, we expected that niche positions at the phylum and species levels are related and also the relationship between the occupancy and abundance of each phylum. Finally, we explored the main factors driving the above water-depth patterns in bacterial phyla and further quantified the relative importance of physical, chemical, and biological drivers.

## Materials and Methods

### Study Region

The studied Lake Lugu, with an elevation of 2,685 m, is located in the Yunnan Province, Southwest China (27°41′–27°45′N, 100°45′–100°50′E). It is one of the deepest freshwater plateau lakes in the region. This lake has spatial characteristics that include high connectivity and a small area, allowing species to be freely distributed at different locations with no apparent barriers to dispersal. Macrophytes were partly absent from the lake, allowing for the collection of surface sediments in shallow waters. In August 2010, we collected 37 surface sediment (~0–1 cm) samples along the water-depth gradient from 0 to 93.5 m. At each site, three sediment cores with a 6-cm diameter were retrieved for surface sediments. The sample collection process was described in detail in a previous study (Wang et al., 2012b).

### Bacterial Community Analysis

Bacterial analyses were performed according to the previous literature (Wang et al., 2012a). Briefly, genomic DNA was extracted from surface sediment samples using the phenol chloroform method (Zhou et al., 1996). Bacterial tag-encoded FLX amplicon pyrosequencing was conducted as described previously (Dowd et al., 2008). Bacterial 16S rRNA genes were amplified using the 27F primer (5′ GAG TTT GAT CNT GGC TCA G 3′) with the 454 Life Sciences A sequencing adapter, and the modified 519R primer (5′ GTN TTA CNG CGG CKG CTG 3′) with a 8-bp barcode sequence and the 454 Life Sciences B sequencing adapter. The PCR amplification of a single 35 cycle was performed in a ABI9700 thermocycler (ABI, Foster City, USA) using the program 95°C for 2 min; 25 cycles of 95°C for 30 s, 55°C for 30 s, 72°C for 30 s; 72°C for 5 min; finally kept at 10°C. Triplicate positive PCR products were pooled and purified with AxyPrep DNA Gel Extraction Kit (Axygen, USA). Amplicon sequencing was performed based upon the manufacturer protocols (Roche Applied Science, Indianapolis, IN) for Titanium sequencing on FLX-titanium platform (Dowd et al., 2008). The purified amplicons were pooled at equal molality, and then sequenced using a Roche 454 FLX pyrosequencer (Roche, Switzerland).

All 16S rRNA pyrosequencing reads were analyzed using QIIME, version 1.9.0 (Caporaso et al., 2010b). We imported barcoded 16S rRNA gene sequences and removed the primers, demultiplexed reads, and then filtered them according to Phred quality scores. Quality criteria were a minimum sequence length of 200 bp, a maximum sequence length of 900 bp, and a minimum average quality score of 25. With such quality control, we allowed few ambiguous bases or mismatches in the primer sequence and no barcode errors, and a maximum homopolymer length of 6 bp.

Sequences were denoised with the Denoiser algorithm (Reeder and Knight, 2010), clustered into operational taxonomic units (OTUs) at 97% similarity level with the seed-based UCLUST algorithm (Edgar, 2010). We used the longest sequence in a cluster as the representative sequence for that OTU. Singletons, i.e., OTUs with only one read in the entire dataset, were removed and chimeras were excluded using ChimeraSlayer (Haas et al., 2011). After chimeras were removed *via* ChimeraSlayer, representative sequences from each OTU were aligned to the Greengenes v13.8 imputed core reference alignment (DeSantis et al., 2006) using PyNAST (Caporaso et al., 2010a). The taxonomic identity of each representative sequence was determined using the RDP Classifier (Wang et al., 2007) and chloroplast and archaeal sequences were removed. Before the subsequent analysis, bacterial community were rarefied at 1,139 sequences to avoid the variation in abundance or sampling intensity biased. The generated sequences can be found in figshare (https://doi.org/10.6084/m9.figshare.8052788).

### Abiotic and Biotic Variables

We evaluated several environmental characteristics important to the biological community in the surface water of 0.5 m and bottom water near the sediment-water interface at each site (Supplementary Table S1). We analyzed the temperature, pH, conductivity, total nitrogen, total phosphorus, dissolved oxygen (DO), ${HCO}_{3}^{-}$ concentration (HCO_3_·water), and silica of the surface water, and measured the temperature, pH, conductivity, and DO of the bottom water. In addition, the surface sediment total phosphorus, loss-on-ignition (LOI), water content, grain size, porosity, and the metal ion concentrations were measured. The measured metal ions included Al, Ba, Be, Ca, Co, Cr, Cu, Fe, Li, K, Mg, Mn, Na, Ni, Pb, Sr., Ti, V, and Zn. The grain size was divided into five classes: <4, 4–16, 16–32, 32–64, and >64 μm. A detailed description of the methods for the measurement and calculation of the abiotic variables was described in a previous study (Wang et al., 2012b).

In terms of the biotic variables, diatoms and chironomids were classified and identified to the lowest possible taxonomic level (typically to the species level) as shown in previous studies (Wang et al., 2012b; Zhang et al., 2013). We used the following biotic factors as predictor variables: (1) the chlorophyll *a* concentration of the surface and bottom water, (2) the biomass of diatoms and chironomids, and (3) the richness of diatoms and chironomids. The concentration of chlorophyll *a* was the representative of the phytoplankton biomass. The biomass of diatoms and chironomids was indicated by the number of diatoms per gram of wet sediment and the count of head capsules per gram of wet sediment, respectively.

### Statistical Analyses

First, we only included the highly abundant bacterial phyla by considering species occurrence percentages larger than 85%. We explored the relationships between water depth and the relative abundance of bacterial phyla with linear or quadratic models. The most appropriate model was selected based on the lowest value of Akaike’s information criterion (Yamaoka et al., 1978).

Second, we calculated the niche position of the water depth for both the species and phylum levels. For each species or phylum, we estimated the weighted water-depth position by averaging the product of their water-depth values and their abundances across all samples (Wang et al., 2013). We selected the linear or quadratic model based on the lower value of Akaike’s information criterion and explored the relationship of niche position between the phylum and species levels. In practice, the proportion of sites was considered as “occupancy,” and the mean abundance at occupied sites was considered as “abundance.” The relationship between the occupancy and abundance of bacterial phyla was examined by linear or quadric models, and the better model was selected based on the lower value of Akaike’s information criterion (Yamaoka et al., 1978).

Third, to identify the important factors affecting each phylum, we applied random forest model (Feld et al., 2016) and redundancy analysis (RDA). Statistical dependence between the explanatory variables was assessed using Pearson’s correlation coefficients, and variables with high correlation coefficients (Pearson *r* > 0.7) were excluded from the models. By performing principal component analysis, the 19 metal ion concentrations were reduced to the first two axes of the principal components analysis, which thus represented the environmental variables that reflected the geochemical factors. This process was performed to decrease the degrees of freedom so that they were lower than the number of sampled sites. The other measured variables were used as environmental variables without principal component analysis. For the random forest analysis, we used techniques such as cross-validation to prune the 2,000 trees to an optimal size (Prasad et al., 2006). The importance of a predictor variable was determined by its frequency of selection weighted by a measure of improvement of the model given each split and averaged across all trees (contributions were scaled to sum to 100). Furthermore, RDA was used to examine the potential explanatory variables of community composition at the phylum level. We used RDA with Hellinger-transformed abundance data of the bacterial community, as this transformation makes complex data with numerous zero values more suitable for the analysis with linear methods (Legendre and Gallagher, 2001).

Finally, we applied variation partitioning to quantify the relative importance of the main environmental drivers using linear model (Anderson and Cribble, 1998; Legendre, 2008). We categorized the explanatory variables into three groups: physical, chemical, and biological components. The physical component included water depth, conductivity of surface water, porosity, and grain size. The chemical component included surface water pH, total phosphorus, total nitrogen, SiO_2_ and HCO_3_, bottom water DO and pH, and surface sediment LOI, grain size, PC1 and PC2 of metals. For biological component, we considered the richness and biomass of diatoms and chironomids, and chlorophyll *a* of surface and bottom water. We generated three explanatory matrix models and estimated the proportions of variation in the relative abundance of the bacterial phyla explained by these three components. For each component, significant explanatory variables were selected by forward selection against the Hellinger-transformed abundance phylum data with 9,999 permutations.

We did not apply more statistical analyses because it is a challenge to differentiate the direct or indirect effects of underlying drivers on the relative abundance of bacterial phyla based on field observations. These above analyses were conducted with vegan V2.5-5 (Oksanen et al., 2013) and randomForestSRC V2.9.0 (Ishwaran and Kogalur 2014) in the R environment.

## Results

**Figure 1:**
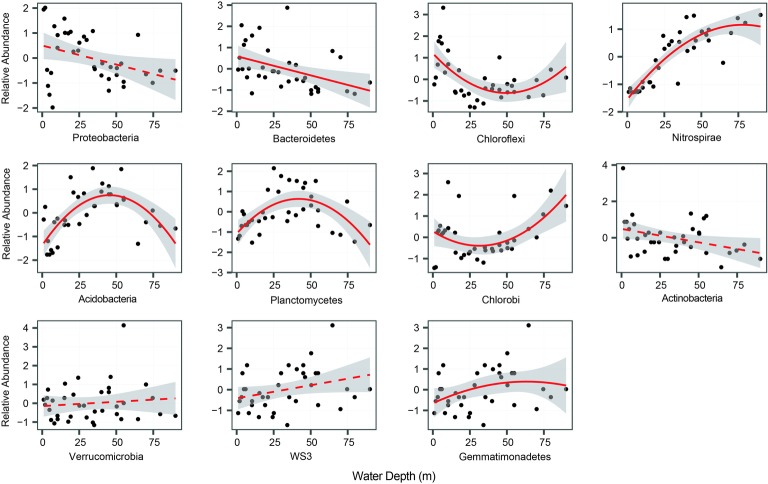
The relative abundance of bacterial phyla along the water-depth gradient. The relationships between water depth and relative abundance were modeled with linear and quadratic models. The better model was selected based on the lower value of Akaike’s information criterion. For better visualization, species relative abundance was scaled as mean = 0 and SD = 1.

In total, we selected 11 major bacterial phyla based on their relative abundance, including Proteobacteria, Nitrospirae, Chlorobi, Chloroflexi, and Bacteroidetes. Among these phyla, Proteobacteria was the most dominant, followed by Bacteroidetes, Chloroflexi, and Nitrospirae (Table 1). The relative abundance of most phyla, seven out of 11, exhibited significant (*p* < 0.05) and clear water-depth patterns (Figure 1), such as increasing, decreasing, hump-shaped, and U-shaped trends. For example, the relative abundance of Bacteroidetes decreased with water depth, while that of Nitrospirae increased toward deep water (Figure 1). In addition, Acidobacteria, Planctomycetes, and Gemmatimonadetes showed hump-shaped trends along the water-depth gradient (Figure 1). Interestingly, the phototrophic phyla Chlorobi and Chloroflexi showed U-shaped patterns.

**Table 1:** Abbreviations for the abundant phyla.

Phyla	Abbrev.	Relative abundance (%)
Proteobacteria	PRO	35.80
Bacteroidetes	BAC	10.06
Chloroflexi	CHF	9.58
Nitrospirae	NIT	8.85
Acidobacteria	ACI	5.39
Planctomycetes	PLA	4.35
Chlorobi	CHB	3.69
Actinobacteria	ACT	3.22
Verrucomicrobia	VER	3.06
WS3	WS3	0.87
Gemmatimonadetes	GEM	0.83

*The Table includes the most abundant bacterial phyla that together contributed up to 85% of the total sequence read. Relative abundance represents the average value of each phylum across all sites*.

**Figure 2:**
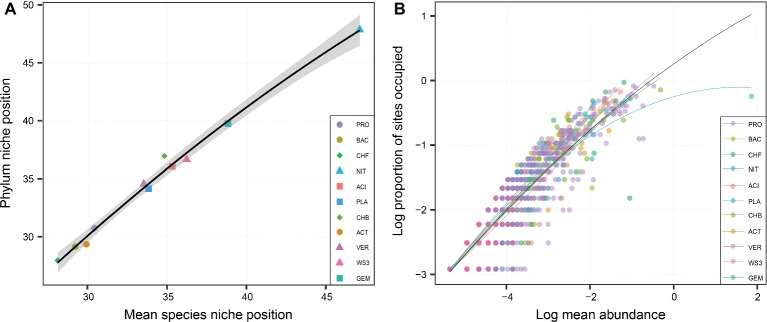
The relationship of niche position between the phylum and species levels **(A)**, and the relationships between the occupancy and abundance of each phylum **(B)**. The shapes in **(A)** represent various distributional patterns: ■, hump-shaped pattern; ●, decreasing pattern; ▲, increasing pattern; ♦, U-shaped pattern. **(B)** Relationships between occupancy and mean local abundance for bacterial phyla. The points in **(B)** represent the different species. Linear or quadratic models were selected according to a lower value of Akaike’s information criterion.

At the phylum level, the niche position of water depth showed great variations, ranging from 26 to 48 m (Figure 2A). The phylum-level niche positions had significant (*p* < 0.05) positive correlations with those at the species level (Figure 2A). For the whole bacterial community, there was a significant relationship between occupancy and abundance (Figure 2B). This is consistent with the finding at the phylum level, which also showed rather strong and positive occupancy-abundance relationships (Figure 2B). However, there were no clear differences among phyla in terms of the relationship of occupancy and mean abundance.

**Figure 3:**
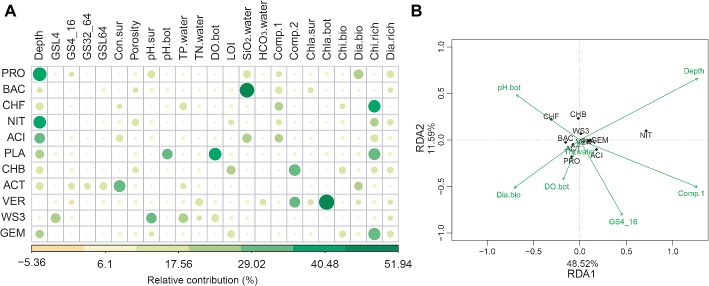
The abiotic and biotic factors related to the relative abundance of bacterial phyla. These factors were identified with random forest **(A)** and redundancy analysis (RDA, **B**). For abiotic factors, we considered water depth (depth), total phosphorus (TP.water), total nitrogen (TN.water), conductivity (Con.sur) and concentrations of ${HCO}_{3}^{-}$ (HCO_3_·water) and SiO_2_ (SiO_2_·water), pH (pH.bot and pH.sur), dissolved oxygen (DO.bot), sediment porosity, loss-on-ignition (LOI), the first two axes of metal ion principal component analysis (Comp.1 and Comp.2) and grain size. Grain size: <4 μm (GSL4), 4–16 μm (GS4_16), 32–64 μm (GS32_64), and > 64 μm (GSL64). Biotic factors included the biomass (Dia.bio and Chi.bio) and richness (Dia.rich and Chi,rich) of diatoms and chironomids, and chlorophyll *a* (Chla.bot and Chla.sur). For RDA, the abiotic variables were automatically selected based on Monte Carlo permutation tests (999 permutations).

The random forest analyses showed that water depth was the strongest variable correlating with the relative abundance of the most bacterial phyla. Other environmental variables, such as metal ion concentrations, SiO_2_, pH, total phosphorus, DO, and conductivity also have important effects on bacterial phyla (Figure 3A). Interestingly, biological factors such as chironomid richness and chlorophyll *a* concentration were also important for some bacterial phyla (Figure 3A). Such findings were confirmed by RDA, which shows that water depth was the strongest factor (*p* < 0.001) related to the distribution of bacterial phyla, while biological attributes, such as diatoms biomass, also showed significant (*p* = 0.038) correlations (Figure 3B).

**Figure 4:**
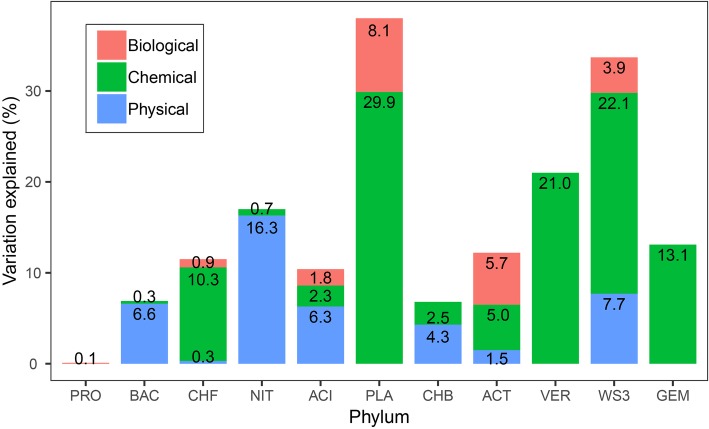
The proportion of the variance in bacterial phylum relative abundance explained by physical, chemical, and biological variables. For simplicity, the pure effects of the three components in predicting the relative abundance are shown, but not the joint effects or unexplained variances. An alternative version of this figure showing the unique and shared variance of each group can be found in Supplementary Figure S1.

In the variation partitioning analyses with the three variable components, the pure effect of chemical component was the higher for bacterial phyla rather than the pure effects of physical and biological components. The relative abundance of most phyla, such as Planctomycetes (29.9%), WS3 (22.1%), and Verrucomicrobia (21.0%), was well explained by the pure effect of chemical component. This is especially true for Verrucomicrobia and Gemmatimonadetes, which were only explained by the pure effect of chemical component (Figure 4). Physical and biological variables had effects on seven and six bacterial phyla, respectively. For Nitrospirae, physical variables were especially important, which explained 16.3% of its variation. The pure effect of biotic variables accounted for 8.1% variations of the relative abundance of Planctomycetes. For Proteobacteria, only 0.1% was explained by biological variables. Four bacterial phyla can be explained by the combination of pure effects of biological, chemical, and physical variables.

## Discussion

A long-standing task in ecology has been to explain biological distribution patterns and the drivers underlying these patterns. In recent years, the water-depth patterns of microbial community distribution have been extensively investigated in aquatic systems. However, most studies have focused on marine environments (Smith and Brown, 2002; Wang et al., 2013), while few studies have been conducted in freshwater ecosystems, such as lake sediments (Haglund et al., 2003; De Wever et al., 2005; Yu et al., 2014). The main aim of our study was to explore the distributional patterns of bacterial abundance at the phylum level and to analyze the abiotic and biotic variables explaining these patterns. Our results highlight four main findings. First, the bacterial phyla exhibited various patterns along the water-depth gradient, some of which are rarely reported, such as the hump-shaped and U-shaped patterns. Second, the occurrence of these patterns across phyla might be explained by the variation in their niche positions, while the occupancy-abundance relationships were not dependent on phylum attributes. Third, among all measured exploratory variables, water depth was the most important predictor of the relative abundance of bacterial phyla. Finally, although the environmental variables were shown to have pivotal roles in terms of the relative abundance of bacterial phyla, biotic variables could also substantially explain the abundance of some phyla, such as Planctomycetes, Actinobacteria, and WS3.

Proteobacteria was the most dominant phyla in the sediments of this lake, followed by Bacteroidetes, Chloroflexi, and Nitrospirae. This is consistent with previous studies that Proteobacteria and Bacteroidetes are the most abundant phyla in sediments (Diao et al., 2017). These phyla have frequently been shown to dominate freshwater bacterial communities (Dai et al., 2016). At present, investigations of the spatial pattern of bacterial relative abundance in freshwater lake sediments indicate that bacterial abundance shows clear spatial variations (Bouzat et al., 2013; Liu et al., 2015a). However, the distributional patterns of bacterial abundance along water-depth gradients, especially among bacterial phyla, have been less explored. Bacterial abundance generally decreases with water depth in freshwater systems (Liu et al., 2016). Our results reveal various water-depth patterns in terms of the relative abundance of bacterial phyla, among which some patterns have been rarely reported so far. For instance, there were significantly (*p* < 0.05) decreasing patterns for Bacteroidetes, increasing patterns for Nitrospirae, and hump-shaped patterns for Acidobacteria, Planctomycetes, and Gemmatimonadetes. The decreasing and increasing water-depth patterns of the relative abundance of bacterial phyla are consistent with the patterns found in earlier studies along other environmental gradients, such as elevations. For instance, the relative abundance of Actinobacteria, Bacteroidetes, and Deltaproteobacteria increases with elevation in stream biofilms, while that of Alphaproteobacteria decreases with elevation (Wang et al., 2012a). However, the U-shaped patterns of Chlorobi and Chloroflexi were unexpected because such patterns are extremely rarely observed in nature, and these two phyla have phototrophic capability (Gupta, 2010). The two phyla are commonly present in the surface waters and bottom waters of deep lakes and oceans (Newton et al., 2011). The phylum Chlorobi is resistant to very low light conditions (Freed et al., 2019) and primarily exists in the anoxic aquatic environment of stratified lakes (Gupta, 2010). Chloroflexi, however, contains both photosynthetic and nonphotosynthetic members (Bryant and Frigaard, 2006), but metabolic insights suggest that a primarily heterotrophic lifestyle boosted by light-driven energy generation *via* aerobic anoxygenic phototrophy occurs in some groups (Mehrshad et al., 2018). It should be noted that alternative explanations may be needed for such U-shaped patterns of Chlorobi and Chloroflexi.

These water-depth patterns may be explained by the niche positions of the bacterial phyla. Consistent with the various water-depth patterns of relative abundance, the niche position also obviously differed among phyla. For instance, the phyla with decreasing water-depth patterns had a lower niche position, while those with increasing and hump-shaped patterns had a higher niche position. In other words, niche position may have a certain impact on bacterial phylum distributional patterns. This phenomenon has also been reported for other taxonomic groups, such as the distribution of stream insects, the predictability of which depends on niche position (Heino and de Mendoza, 2016). Furthermore, niche position could be an important predictor of the regional occupancy and local abundance of diatoms and insects, such as those in streams (Rocha et al., 2018). However, the occupancy-abundance relationship showed a relatively similar trend for all phyla. This result may have occurred because of the taxonomic relatedness among bacteria, as taxonomic relatedness has only minor influence on occupancy and abundance, as observed among the diatoms and macroinvertebrates across a set of lakes (Heino and Tolonen, 2018). Hence, the occupancy-abundance relationship could not explain the variation in water-depth patterns across phyla.

Physicochemical variables were important in explaining the relative abundance of bacterial phyla along the water-depth gradient. Among them, water depth was the most important environmental factor affecting the relative abundance of bacterial phyla in this lake ecosystem, which is consistent with previous studies showing that water depth has important effects on bacterial community composition (Yokokawa et al., 2010; Zhang et al., 2015). However, water depth could also be associated with many other physiochemical variables, including temperature, pressure, productivity, and nutrient availability (Smith and Brown, 2002; Bryant et al., 2012). It seems that water depth is only a proxy for multiple physiochemical variables, and we cannot completely exclude the influence of other environmental and biological factors on the vertical distribution of bacterial phyla.

In addition, some other environmental factors, such as metal ion concentrations, SiO_2_, pH, total phosphorus, and conductivity, were also related to some bacterial phyla abundance in our study. This is in line with the finding that species are filtered by environmental factors and reproduce more under more suitable conditions (Schei et al., 2012; Alofs and Jackson, 2015). For instance, sediment bacterial abundance in freshwater plateau lakes could be influenced by pH and conductivity (Xiong et al., 2012; Liu et al., 2016), and ion concentrations are an important factor controlling bacterial community composition in lake system (Liu et al., 2014). Environmental variables, such as pH and silicate silicon, are important for the composition of the bacterial community in lake sediments, such as those in an Arctic lake area (Wang et al., 2016).

However, we also found that biotic variables were important in accounting for the relative abundance of bacterial phyla. For example, biotic variables explained 8.1, 5.7, 3.9, and 1.8% of the variation in Planctomycetes, Actinobacteria, WS3, and Acidobacteria, respectively. In particular, the relative abundance of Proteobacteria was only explained by biological variables of 0.1%, indicating that additional abiotic and biotic interactions are involved. Biotic variables, such as chlorophyll *a*, and chironomid richness, were identified to be important for the abundance of some bacterial phyla. This is in line with previous findings that biotic interactions can affect species distributions and alter biodiversity patterns (Alofs and Jackson, 2015; Sarker et al., 2018). Bacterial abundances within the lakes are positively correlated with chlorophyll *a* (Lee and Bong, 2008; Liu et al., 2016). In aquatic ecosystems, phytoplankton excrete dissolved organic matter, such as extracellular organic carbon, which may be an important source of bacterial growth. Phytoplankton and bacteria also compete for common inorganic phosphorus resources (Aota and Nakajima, 2001). Bacteria and benthic diatoms have a mutually beneficial effect in terms of promoting microalgae growth and maintaining the bacterial community (Jauffrais et al., 2017). For example, in marine environments, the bacterial abundance and community structure largely depend on the growth and physiological status of diatoms (Grossart et al., 2005), and bacteria can also control algal populations by inhibiting the growth of diatoms or by the active lysis of algal cells (Paul and Pohnert, 2011). Moreover, experimental evidence has shown that bacterial communities and chironomid larvae have a symbiotic relationship (Kuncham et al., 2017), chironomids can influence bacterial densities by grazing, and bacterial densities can in turn be related to variation in larval chironomids density (Schmid and Schmid-Araya, 2010).

## Conclusion

In summary, we found that the relative abundance of bacterial phyla showed various water-depth patterns, such as hump-shaped and U-shaped trends. The niche position of each phylum was significantly different, which is consistent with their abundance distribution patterns. The occupancy-abundance relationship did not show differences among phyla, and niche position could be one of the predictors of the distribution of bacterial phyla. We further found that water depth was the predominant driver of the relative abundance of bacterial phyla. The relative abundance was influenced mainly by chemical variables, while the pure effect of biological variables was also important for some phyla, such as Planctomycetes, Actinobacteria, and WS3. We provided new evidence regarding the important role of biological variables in explaining the variation in bacterial communities from the perspective of the relative abundance of bacterial phyla.

## Data Availability

The datasets generated for this study can be found in figshare, https://doi.org/10.6084/m9.figshare.8052788.

## Author Contributions

JW conceived the idea and performed the bioinformatic analyses. JW, XY, and QW provided physiochemical and biological data. KW led the statistical analyses with the contributions from WZ and JW and wrote the first draft of the manuscript. KW, JW, and WZ finished the manuscript with the contributions from JS, LZ, and XC. All the authors contributed substantially to the study.

### Conflict of Interest Statement

The authors declare that the research was conducted in the absence of any commercial or financial relationships that could be construed as a potential conflict of interest.
