# Supplementary material for: The Relative Abundance of Benthic Bacterial Phyla Along a Water-Depth Gradient in a Plateau Lake: Physical, Chemical, and Biotic Drivers
Source: Front Microbiol. 2019 Jul 10;10:1521. doi: 10.3389/fmicb.2019.01521 (PMC6635551; doi:10.3389/fmicb.2019.01521)
Supplement: Supplementary file 1 [file Data_Sheet_1.docx]

**Supplementary materials**

**The relative abundance of benthic bacterial phyla along a water-depth gradient in a plateau lake: physical, chemical and biotic drivers**

Kaiyuan Wu^a,b,#^, Wenqian Zhao^b,c,^^#^, Qian Wang^b^, Xiangdong Yang^b^, Lifeng Zhu^c^, Ji Shen^b^, Xiaoying Cheng^a,*^, Jianjun Wang^b,d,*^

^a^ School of Environment and Civil Engineering, Jiangnan University, Wuxi 214122, China

^b^ State Key Laboratory of Lake Science and Environment, Nanjing Institute of Geography and Limnology, Chinese Academy of Sciences, Nanjing 210008, China

^c^ School of Biological Sciences, Nanjing Normal University, Nanjing 210046, China

^d^ University of Chinese Academy of Sciences, Beijing 100049, China

^#^ These authors contributed equally to this work.

* Correspondence:

Jianjun Wang, [jjwang@niglas.ac.cn](mailto:jjwang@niglas.ac.cn); Xiaoying Cheng, [chengxiaoysytu@163.com](mailto:chengxiaoysytu@163.com)

**Running title**: Water-depth patterns of bacteria phyla

**Table S1.** **Environmental characteristics important to benthic bacterial phyla**. Depth (m): water depth. pH.bot: pH of bottom water. DO.bot (mg/L): dissolved oxygen of bottom water. Con.sur (μS.cm^-2^): conductivity of surface water. pH.sur: pH of surface water. TP.water (μg/L): total phosphorus of surface water. TN.water (μg/L): total nitrogen of surface water. SiO_2_.water (mg/L): SiO_2_ of surface water. HCO_3_.water (mg/L): concentration of HCO_3_-. pH.bot: pH of bottom water. GSL4: grain size less than 4 µm in surface sediments. GS4-16: grain size 4-16 µm in surface sediments. GS32-64: grain size 32-64 µm in surface sediments. GSL64: grain size more than 64 µm in surface sediments. LOI (%): loss-on-ignition in surface sediment. Porosity (%): porosity in surface sediment. Comp1 and Comp2: the first and second axes of a principal component analysis for 19 metal ions concentrations in sediments, such as Al, Ba, Be and Ca. Environmental factors with Pearson’s correlation less than 0.7 were shown.

|  | Longitude | Latitude | Depth | pH.bot | DO.bot | Con.sur | pH.sur |
| --- | --- | --- | --- | --- | --- | --- | --- |
| 01 | 100.771 | 27.684 | 1.0 | 9.1 | 6.2 | 219 | 9.1 |
| 02 | 100.771 | 27.684 | 2.0 | 9.2 | 9.0 | 205 | 9.2 |
| 03 | 100.771 | 27.684 | 3.3 | 9.2 | 9.3 | 203 | 9.2 |
| 04 | 100.771 | 27.684 | 3.9 | 9.0 | 7.0 | 217 | 9.0 |
| 05 | 100.771 | 27.684 | 4.9 | 9.0 | 7.1 | 217 | 9.0 |
| 06 | 100.772 | 27.684 | 6.0 | 9.1 | 6.9 | 218 | 9.1 |
| 07 | 100.773 | 27.684 | 7.0 | 9.1 | 6.9 | 218 | 9.1 |
| 08 | 100.771 | 27.685 | 8.2 | 9.1 | 6.9 | 217 | 9.1 |
| 09 | 100.815 | 27.739 | 10.3 | 9.0 | 7.0 | 209 | 9.0 |
| 10 | 100.778 | 27.67 | 10.4 | 9.1 | 6.3 | 218 | 9.2 |
| 11 | 100.814 | 27.675 | 15.0 | 9.0 | 7.8 | 217 | 9.2 |
| 12 | 100.814 | 27.741 | 15.4 | 8.9 | 6.8 | 209 | 9.0 |
| 13 | 100.814 | 27.675 | 17.6 | 8.9 | 7.6 | 205 | 9.2 |
| 14 | 100.825 | 27.682 | 19.2 | 8.7 | 6.7 | 203 | 9.0 |
| 15 | 100.812 | 27.738 | 21.1 | 9.0 | 8.3 | 209 | 9.0 |
| 16 | 100.811 | 27.734 | 24.3 | 8.9 | 7.8 | 208 | 9.0 |
| 17 | 100.825 | 27.683 | 25.1 | 8.6 | 6.9 | 207 | 9.2 |
| 18 | 100.824 | 27.681 | 28.2 | 8.9 | 7.8 | 208 | 9.0 |
| 19 | 100.81 | 27.731 | 30.0 | 8.8 | 7.2 | 209 | 9.0 |
| 20 | 100.82 | 27.68 | 34.2 | 9.1 | 6.7 | 208 | 9.2 |
| 21 | 100.761 | 27.736 | 35.1 | 8.9 | 7.9 | 208 | 9.1 |
| 22 | 100.808 | 27.732 | 35.5 | 8.8 | 7.2 | 209 | 9.1 |
| 23 | 100.814 | 27.678 | 40.0 | 8.8 | 7.4 | 207 | 9.0 |
| 24 | 100.807 | 27.73 | 40.6 | 8.9 | 7.2 | 208 | 9.0 |
| 25 | 100.781 | 27.675 | 45.0 | 9.0 | 5.1 | 207 | 8.7 |
| 26 | 100.807 | 27.732 | 45.0 | 8.9 | 7.3 | 208 | 9.0 |
| 27 | 100.778 | 27.682 | 46.2 | 9.0 | 7.0 | 208 | 9.1 |
| 28 | 100.804 | 27.732 | 50.3 | 8.9 | 7.2 | 209 | 9.0 |
| 29 | 100.754 | 27.727 | 50.4 | 8.9 | 7.0 | 209 | 9.1 |
| 30 | 100.779 | 27.683 | 53.4 | 9.0 | 7.0 | 209 | 9.0 |
| 31 | 100.774 | 27.686 | 55.0 | 9.0 | 7.0 | 210 | 9.1 |
| 32 | 100.799 | 27.729 | 55.0 | 8.9 | 7.0 | 209 | 9.0 |
| 33 | 100.775 | 27.696 | 64.7 | 8.9 | 3.9 | 209 | 8.7 |
| 34 | 100.773 | 27.7 | 70.1 | 9.0 | 6.8 | 209 | 9.0 |
| 35 | 100.772 | 27.71 | 74.6 | 9.2 | 6.6 | 210 | 9.1 |
| 36 | 100.773 | 27.711 | 79.7 | 9.2 | 6.0 | 208 | 9.2 |
| 37 | 100.773 | 27.711 | 90.0 | 9.1 | 6.4 | 205 | 9.2 |

|  | TP.water | TN.water | SiO_2_.water | HCO_3_.water | GSL4 | GS4_16 |
| --- | --- | --- | --- | --- | --- | --- |
| 01 | 76.9 | 116.0 | 0.9 | 73.2 | 19.5 | 28.8 |
| 02 | 54.8 | 136.4 | 0.8 | 85.4 | 30.3 | 41.4 |
| 03 | 44.4 | 156.2 | 0.5 | 73.2 | 28.0 | 35.2 |
| 04 | 74.1 | 128.5 | 0.6 | 36.6 | 28.8 | 33.6 |
| 05 | 63.4 | 122.7 | 0.6 | 122.0 | 30.2 | 36.9 |
| 06 | 49.5 | 129.6 | 0.5 | 48.8 | 36.2 | 37.9 |
| 07 | 87.3 | 109.8 | 0.6 | 109.8 | 42.5 | 32.7 |
| 08 | 83.8 | 104.6 | 0.9 | 97.6 | 36.9 | 40.2 |
| 09 | 41.0 | 209.3 | 0.6 | 73.2 | 42.3 | 36.4 |
| 10 | 51.6 | 231.7 | 0.4 | 73.2 | 34.7 | 41.8 |
| 11 | 44.6 | 184.7 | 0.9 | 85.4 | 37.2 | 40.2 |
| 12 | 41.0 | 88.0 | 0.5 | 73.2 | 37.4 | 47.8 |
| 13 | 44.6 | 172.1 | 1.2 | 85.4 | 28.4 | 34.0 |
| 14 | 48.1 | 192.0 | 0.7 | 122.0 | 45.4 | 47.6 |
| 15 | 42.8 | 128.1 | 0.5 | 73.2 | 30.8 | 45.3 |
| 16 | 42.7 | 105.4 | 0.9 | 73.2 | 23.7 | 49.5 |
| 17 | 46.3 | 108.2 | 0.9 | 73.2 | 40.6 | 48.1 |
| 18 | 46.3 | 215.3 | 0.9 | 36.6 | 18.5 | 54.6 |
| 19 | 44.6 | 118.6 | 0.9 | 73.2 | 33.2 | 42.1 |
| 20 | 53.4 | 136.8 | 1.8 | 73.2 | 29.7 | 53.7 |
| 21 | 42.8 | 124.9 | 0.6 | 97.6 | 39.8 | 36.8 |
| 22 | 53.1 | 146.2 | 0.8 | 48.8 | 14.1 | 39.5 |
| 23 | 46.3 | 120.1 | 1.0 | 61.0 | 30.3 | 55.1 |
| 24 | 44.6 | 139.6 | 0.7 | 73.2 | 39.3 | 31.5 |
| 25 | 49.9 | 80.4 | 0.8 | 61.0 | 19.5 | 53.8 |
| 26 | 65.7 | 154.4 | 0.9 | 61.0 | 50.0 | 41.1 |
| 27 | 44.6 | 187.2 | 0.9 | 73.2 | 34.7 | 40.5 |
| 28 | 48.6 | 117.2 | 0.5 | 85.4 | 53.0 | 32.8 |
| 29 | 42.8 | 87.7 | 0.4 | 158.7 | 31.3 | 40.3 |
| 30 | 48.1 | 309.4 | 1.7 | 73.2 | 23.1 | 39.1 |
| 31 | 41.0 | 107.7 | 0.7 | 48.8 | 48.2 | 38.6 |
| 32 | 47.0 | 119.5 | 0.5 | 73.2 | 31.0 | 41.0 |
| 33 | 18.5 | 260.5 | 1.2 | 73.2 | 48.4 | 36.9 |
| 34 | 42.8 | 127.8 | 0.9 | 109.8 | 35.5 | 40.9 |
| 35 | 42.8 | 85.8 | 1.2 | 85.4 | 19.1 | 35.7 |
| 36 | 41.0 | 193.3 | 0.5 | 73.2 | 45.8 | 38.2 |
| 37 | 49.9 | 157.2 | 0.5 | 109.8 | 17.9 | 31.4 |

|  | GS32_64 | GSL64 | LOI | Porosity | Comp.1 | Comp.2 |
| --- | --- | --- | --- | --- | --- | --- |
| 01 | 20.0 | 15.6 | 8.5 | 70.1 | 9.2 | 8.2 |
| 02 | 8.9 | 3.9 | 12.9 | 66.9 | 2.1 | 9.1 |
| 03 | 10.6 | 10.3 | 22.7 | 67.9 | 1.2 | 9.0 |
| 04 | 12.8 | 8.2 | 7.4 | 65.3 | 1.2 | 9.0 |
| 05 | 9.9 | 6.3 | 6.4 | 64.8 | 1.0 | 9.0 |
| 06 | 8.0 | 1.7 | 7.5 | 69.8 | 1.3 | 9.0 |
| 07 | 8.9 | 3.6 | 11.9 | 70.2 | 2.2 | 9.2 |
| 08 | 6.3 | 2.3 | 5.8 | 71.9 | 1.8 | 8.9 |
| 09 | 5.4 | 5.6 | 8.3 | 58.1 | 9.8 | 11.2 |
| 10 | 6.2 | 0.8 | 7.9 | 73.6 | 4.2 | 9.3 |
| 11 | 7.0 | 1.5 | 8.2 | 71.0 | 12.3 | 10.8 |
| 12 | 2.8 | 0.2 | 8.4 | 53.5 | 12.6 | 10.0 |
| 13 | 14.9 | 4.2 | 8.4 | 62.4 | 11.8 | 11.0 |
| 14 | 0.6 | 0.1 | 10.4 | 60.5 | 12.1 | 9.7 |
| 15 | 4.9 | 7.4 | 8.0 | 60.4 | 10.2 | 10.6 |
| 16 | 6.5 | 1.1 | 10.4 | 75.2 | 9.9 | 10.6 |
| 17 | 2.2 | 0.7 | 10.2 | 71.6 | 11.6 | 9.5 |
| 18 | 4.3 | 1.7 | 10.7 | 73.8 | 12.1 | 9.6 |
| 19 | 5.7 | 1.7 | 13.3 | 74.5 | 11.4 | 9.3 |
| 20 | 2.6 | 0.5 | 11.3 | 64.3 | 12.0 | 9.7 |
| 21 | 7.3 | 2.8 | 15.4 | 71.7 | 11.2 | 9.9 |
| 22 | 14.9 | 3.4 | 11.5 | 79.0 | 11.3 | 8.8 |
| 23 | 2.1 | 0.5 | 10.4 | 71.1 | 11.8 | 9.9 |
| 24 | 8.2 | 11.6 | 8.9 | 77.4 | 8.2 | 10.0 |
| 25 | 4.9 | 0.3 | 14.9 | 64.6 | 11.1 | 8.9 |
| 26 | 2.1 | 1.8 | 11.7 | 77.8 | 11.4 | 9.2 |
| 27 | 6.9 | 2.1 | 14.0 | 71.7 | 10.8 | 6.5 |
| 28 | 4.7 | 1.3 | 13.2 | 74.9 | 11.5 | 9.1 |
| 29 | 8.1 | 1.1 | 15.3 | 77.5 | 10.8 | 7.6 |
| 30 | 13.1 | 2.2 | 15.5 | 69.1 | 11.2 | 4.9 |
| 31 | 3.7 | 0.7 | 13.7 | 72.8 | 11.5 | 7.6 |
| 32 | 7.9 | 2.7 | 15.6 | 71.9 | 10.5 | 9.3 |
| 33 | 4.0 | 0.6 | 15.3 | 71.7 | 9.9 | 8.6 |
| 34 | 7.3 | 1.6 | 15.3 | 76.3 | 10.1 | 7.8 |
| 35 | 16.1 | 2.8 | 15.7 | 75.5 | 11.1 | 6.9 |
| 36 | 3.8 | 1.5 | 16.2 | 81.0 | 11.0 | 8.1 |
| 37 | 19.8 | 2.7 | 16.0 | 81.7 | 10.8 | 8.4 |


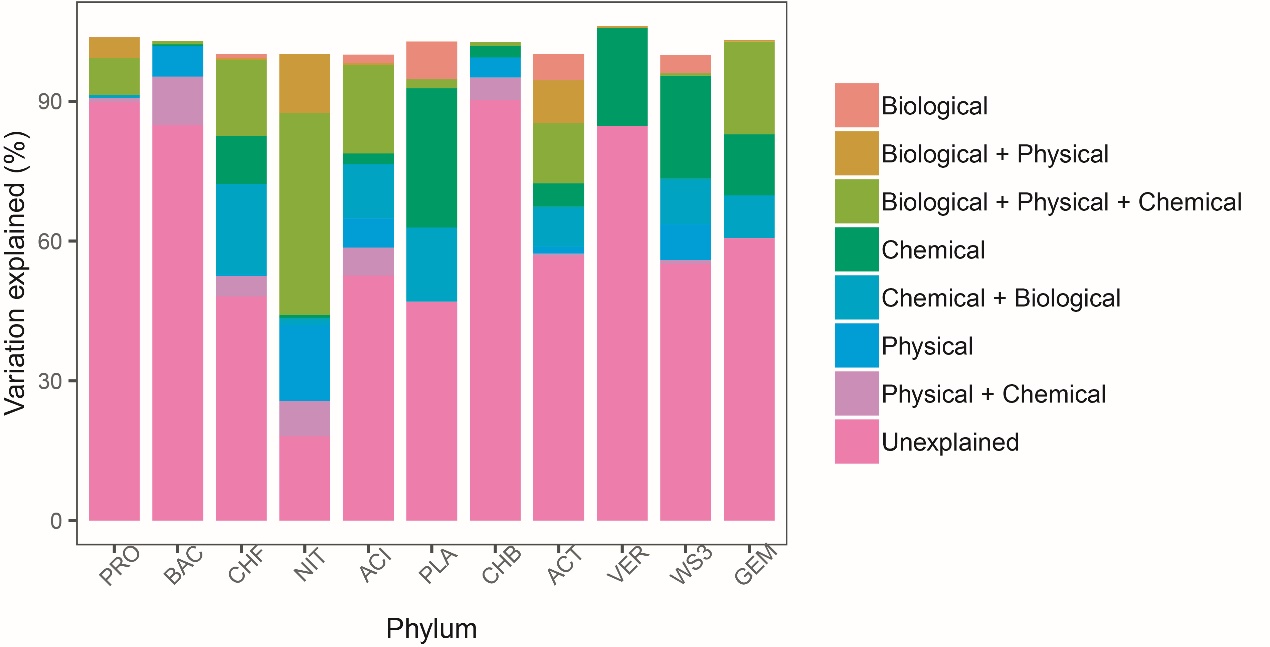


**Figure S1**. **The relative importance of physical, chemical and biological variables in explaining the relative abundance of bacteria phyla**. Only >0% portions of explained variation are plotted.
